# “We Dance and Find Each Other”^1^: Effects of Dance/Movement Therapy on Negative Symptoms in Autism Spectrum Disorder

**DOI:** 10.3390/bs6040024

**Published:** 2016-11-10

**Authors:** Malin K. Hildebrandt, Sabine C. Koch, Thomas Fuchs

**Affiliations:** 1 Department of Psychology, University of Heidelberg, Hauptstr. 48-52, 69117 Heidelberg, Germany; 2 Department of Creative Arts Therapies and Therapy Sciences, Alanus University, Villestr. 3, 53347 Alfter/Bonn, Germany; 3 Department of Therapy Sciences, SRH University Heidelberg, Maria-Probst-Str. 3, 69123 Heidelberg, Germany; 4 Department of General Psychiatry, University of Heidelberg, Vossstr. 2-4, 69115 Heidelberg, Germany; thomas.fuchs@med.uni-heidelberg.de

**Keywords:** autism spectrum disorder, embodiment, negative symptoms, dance movement therapy, randomized control trial, intervention methods

## Abstract

The treatment of deficits in social interaction, a shared symptom cluster in persons with schizophrenia (negative symptoms) and autism spectrum disorder (DSM-5 A-criterion), has so far remained widely unsuccessful in common approaches of psychotherapy. The alternative approach of *embodiment* brings to focus body-oriented intervention methods based on a theoretic framework that explains the disorders on a more basic level than common theory of mind approaches. The randomized controlled trial at hand investigated the effects of a 10-week manualized dance and movement therapy intervention on negative symptoms in participants with autism spectrum disorder. Although the observed effects failed to reach significance at the conventional 0.05 threshold, possibly due to an undersized sample, an encouraging trend towards stronger symptom reduction in the treatment group for overall negative symptoms and for almost all subtypes was found at the 0.10-level. Effect sizes were small but clinically meaningful, and the resulting patterns were in accordance with theoretical expectations. The study at hand contributes to finding an effective treatment approach for autism spectrum disorder in accordance with the notion of *embodiment*.

## 1. Introduction

Autism spectrum disorders (ASDs) and schizophrenia are complex diseases with poorly defined boundaries that have been shown to display a certain overlap of behavioral and cognitive symptoms [1]. This overlap is mainly based on the conceptual relatedness of negative symptoms (NS) in schizophrenia and the social component of autistic symptoms. In DSM-5, NS are divided into the sub-domains of expressive deficits and avolition/asociality, a structure that has been confirmed by factor analysis [2]. Very similar sub-domains are also listed in the A-criterion of ASD as deficits in social communication and social interaction, thus it appears that both symptom clusters are cognate. The relationship between NS and ASD has been phenomenologically demonstrated by Konstantareas and Hewitt [3], who found that considerably more participants with ASD showed negative rather than positive symptoms. Conversely, participants with schizophrenia tended to show only those symptoms of ASD that were related to NS. Only the focus on anhedonia in schizophrenia and on strong deficits in nonverbal reciprocity in ASD differentiate clearly between NS and the DSM-5 A-criterion of ASD. At the level of the disorders taken as a whole, the remaining confining symptoms are repetitive behavior in autism and positive symptoms, such as delusions or hallucinations, in schizophrenia [4]. Unsatisfyingly, in the light of their strong association with poor quality of life, NS have so far remained mostly resistant to conventional treatment methods such as medication and common psychotherapy [5] and the same holds for social functioning in ASD [6,7]. Therefore, interest in and acceptance of alternative treatment methods has risen in the clinical and the scientific community in the past years, spurring research on how to better address the therapeutic challenges of this symptom cluster. 

### 1.1. The Development of Social Cognition

Traditionally, problems in perspective taking and empathy, which represent a fundamental impairment in social interaction, are understood to be the consequence of a deficient Theory of Mind (ToM), found both in ASD and in schizophrenia [8]. This notion assumes that we cognitively derive intentions and emotions underlying other people’s actions. Within that theoretical framework there are two competing views, namely the Theory Theory and the Simulation Theory (ST). According to the Theory Theory we build implicit theories on the co-occurrence of behavior and mental states and how they influence each other. Relying on those implicit theories we then infer mental states from observed behavior and predict future behavior. The Simulation Theory assumes that ToM originates from imagining how we would feel and what we would do in a given situation and attributing this to the person of interest. Although there is disagreement about its underlying mechanism, both approaches insist that a ToM is a necessary condition of social cognition [9]. 

In contrast, invoking research on the Mirror Neuron System (MNS), the *embodied simulation* theory claims that we draw conclusions about others’ mental states by neural mirroring of their actions and matching this representation with memorized mental states related to similar activation patterns. That is, mirror neurons are likewise activated when we perform or observe a certain intentional action, thereby allowing us to *bodily* simulate it and hence *empathize* with the agent and thus understand related intentions and emotions [10,11]. Supporting this notion, research on mimicry has shown that typically developing individuals automatically demonstrate subtle unconscious physical imitation in social interaction [12]. This is possibly due to the activation in the motor areas caused by the MNS and leads to an even stronger connection with corresponding mental states.

### 1.2. Social Cognition in Autism Spectrum Disorder

Individuals with ASD display abnormalities on all levels of this presumed process. First, they demonstrate problems with several non-social motor coordination tasks [12], which might handicap the development of links between the activation patterns of those motor movements and other information (such as emotions or intentions) in the first place. Furthermore, it has been shown that, when compared to healthy controls, participants with ASD show delayed and weaker activation of MNS during imitation of lip postures and a weaker increase in motor-evoked potentials during action observation [13,14], suggesting less neuronal mirroring. Regarding the process of connecting executed or mirrored motor activity patterns with mental states, the decreased connectivity between distant brain regions that has been reported for individuals with ASD could be a limiting factor. For example, decreased connectivity between the cerebellum, which is involved in motor activities, and the thalamus, which distributes sensory input throughout the cortex, has been reported in research on autism [15]. Whereas they may be able to consciously imitate others, individuals with ASD show less automatic mimicry [12]. Although evidence is not unambiguous [8] and cannot yet answer the question as to whether a deficient MNS is causative of the lack of social understanding and motivation or vice versa, it does demonstrate that the ability to physically simulate others actions might be closely related to the ability to understand their emotions and mental states. Supporting this hypothesis on a behavioral level, a study following toddlers diagnosed at risk for ASD found motor skills at age two to be the best predictor for optimal outcome (i.e., not meeting the criteria for ASD anymore) at age four [16].

### 1.3. An Embodied Approach to Autism Spectrum Disorder

In accordance with these findings, but going beyond embodied simulation theory, the interdisciplinary *embodiment* or *embodied cognition* approach assumes that cognition in general is essentially grounded in bodily states [17]. Our perception of the world, and thus also our interaction with it, is entirely mediated by our bodies. For example, if we perceive an object, by whichever sensual modality, this perception becomes apparent to us by reactions of receptors, indicating a change to the “unstimulated” baseline state. Thus, all perceptions are at the same time self-affections and necessarily self-referential. Furthermore, perception leads to an awareness of one’s own body movement in the environment. Our experience of the world results from this self-referential interaction with our surroundings characterized by perception and movement. Hence any changes in normal functioning of the connection of our perception (e.g., visually observing social cues) and movement via our brain (e.g., the activated mirror neurons by observing social cues) have an influence on our experience of the world [18] and therefore on our mind. Rather than solely focusing on the causative influence of the mind on the body, this notion highlights the reciprocal conjunction of both entities, thereby providing a new theoretical starting point for therapeutic interventions. 

Within the *embodiment approach,* underpinned by a phenomenological perspective, ASD is understood as a disorder of self-perception and social interaction, especially empathy, and is hence, in summary, a disorder of intersubjectivity. Gallagher’s *interaction theory of autism* [19] furthermore enumerates problems with *primary intersubjectivity*, the very early developing ability to understand others’ feelings and intentions only by perceiving their body expressions, as the core of ASD (see also [20,21]). Specifying this in greater detail, Mundy et al. [22] argue that these problems might derive from reduced parallel processing of sensory information regarding self (interoception or proprioception), and others (exteroception). This parallel processing is crucial in order to connect information of one’s own bodily status and emotions or intentions with information about the social counterpart and thus for the development of empathy. Hence, difficulties with the very basic social skills of self-perception and/or parallel processing, disrupting *primary intersubjectivity*, might provoke a number of cascading difficulties, constituting the social symptom cluster of ASD.

### 1.4. Embodied Approaches to Autism Therapy

Taking the theoretic approaches and empirical findings explained above seriously, autism therapy should focus more on non-verbal interventions to reinforce the mechanisms underlying primary intersubjectivity. Theories such as Eberhard-Kaechele’s developmental mirroring taxonomy ([23,24], see also Appendix B) and embodied parental mentalizing [25], show how movement and mirroring is intricately connected to cognitive achievements such as mentalization; those theories help bridge mirroring and mentalization theories, and explain how the here applied dynamic therapeutic intervention is expected to inform the social cognitive states. 

Non-conventional therapy approaches such as dance movement therapy (DMT) correspond to the theoretical implications of embodiment, highlighting the role of motor movement and sensorimotor experience for mental states and as a medium for therapeutic change. Yet, embodiment is a theoretical construct and not a psychotherapeutic method, and while embodiment approaches provide a rich and sound theoretical framework for psychotherapy (e.g., [26]), they are not sufficient for an adequate understanding of all the aspects of the therapeutic approaches falling into their domain. DMT as one of the creative arts therapies goes beyond embodiment approaches, for example, into the domain of aesthetics. While DMT mostly takes place in group settings, DMT for children with autism is rather conducted in one-on-one settings [27]. As a conclusion of the presumed process underlying social cognition explained above, the mode of action of DMT on social difficulties in autism can be the following: By practicing the bodily aspects of the process (e.g., mirroring) while at the same time emphasizing one’s own and others’ mental states (increase of parallel processing) the connection of bodily and mental states within the individual is strengthened. Thereby information about one’s own and others mental states becomes more easily accessible when certain bodily states are experienced or observed and thus the basis of empathy is improved. Beyond that, the intervention also enhances self-perception and taken together this adds to improvements in all aspects of the social symptom cluster including affect. Thus, DMT aims to “engage the brain via the body” [28] (p. 213).

So far, evidence indicating positive effects of embodied therapies on individuals with ASD consists mostly of developmental improvements from case-studies [28,29,30,31,32]. For example, Devereaux [30] describes teachers’ comments on children’s improvements after a year of weekly DMT sessions that included increase of eye contact, verbalization, self-awareness and empathy. Adler [33] documented two children’s developmental and interactional improvements during mirroring-based DMT in her classic video documentation “Looking for me” available through the American Dance Therapy Association (www.adta.org). There is one quantitative intervention study demonstrating beneficial effects of DMT on children with autism [34], and one study on adults with ASD [35] namely the feasibility study for this trial. Hartshorne and colleagues [34] showed that 38 children with autism (ages 3–7), after receiving bi-weekly movement therapy in small groups of 3–8 kids led by a trained movement therapist, spent less time wandering, more time showing on-task behavior, less time showing negative responses to being touched, and less time resisting the teacher compared to a matched control group without movement therapy. The feasibility study of our research group [35] showed that after seven sessions of manualized dance movement therapy based on mirroring, young adults with ASD reported improved body awareness, well-being (particularly decreased tension), increased self-other distinction and increased social competence compared to a matched control group of the same rehabilitation institution without the intervention [27].

Regarding schizophrenia, there are three quantitative studies reporting a significant reduction of NS after a body-oriented treatment compared to a control group that received either supportive counseling or treatment as usual [36,37,38], one of which is part of the same research project as the present study. With regard to the conceptual relatedness of NS and symptoms of social impairment in autism and the proposed mode of action from body to mind we hypothesized that manualized movement therapy (based on DMT) would reduce NS in autism.

## 2. Materials and Methods

### 2.1. Study Design

As part of the EU research project “Toward an Embodied Science of Intersubjectivity” (TESIS), the Department of General Psychiatry in Heidelberg, Germany, conducted a study on embodiment approaches on autism and schizophrenia. Two randomized controlled studies engaged in the effects of manualized movement therapy on several aspects of the disorders and was approved by the ethics committee of the medical faculty of the University of Heidelberg. The aim of the study at hand is to examine the effect of this therapy on NS in participants with ASD. A double-blind, two factorial design comprising the factors *Group* (treatment versus control group) and *Time* (before versus after the treatment) was applied. 

### 2.2. Recruitment Procedure and Randomization

Participants were recruited from three therapeutic or rehabilitative facilities specialized in ASD. The inclusion criteria were (1) German as native language or schooling in German since primary school age; (2) age between 14 and 65 and (3) diagnosis of an autism spectrum disorder (International Classification of Disorders (ICD)-10: F84.0, F84.1, F84.5, F84.9). Exclusion criteria were (1) acute psychosis; (2) history of severe brain trauma, neurological, or internist diseases affecting motor abilities; (3) substance abuse or substance-induced psychosis; and (4) IQ < 70. A total of 78 individuals with ASD met the criteria and agreed to participate in the study. They were given detailed information about the study and their right to withdraw from participation at any time. They signed the written informed consent either themselves, or for participants under age parents/custodians consented in writing. All participants received an expense allowance of 20 Euros after the completion of data acquisition.

The treatment was subdivided into three consecutive rounds within a two-year timeframe with six therapy groups of at most ten participants each. Participants were randomly assigned to one of the three groups, one of which formed the waiting control group. The assignment ratio of treatment and control group was intended to be 2:1. However, some participants participated in the control group assessment without subsequently taking part in the treatment. Data on the same variables, except for a questionnaire on experiences in the movement therapy only assessed in the treatment group, was gathered for both groups at the same time interval, forming pre- and post-test. Participants formerly assigned to the waiting control group participated in data assessment again before and after the intervention. A follow up time of six months was intended and partially conducted but did not provide enough data to be included in the analysis. Data assessment was conducted by medical doctors, psychologists and psychology students trained on the used assessment instruments and blind to group affiliation of the study.

### 2.3. Intervention

Participants in the treatment group received ten weekly sessions of manualized dance movement therapy (DMT) while participants in the control group continued with their individual routine that did not comprise other forms of psychotherapy. The sessions took place weekly at the same afternoon hour each week in a facility room of approx. 60 m^2^. Each DMT session took 60 min and consisted of three mirroring exercises and one verbal processing element (for a schematic description see Appendix B). 

As an opening turn the Chace-Circle (about 10 min) [39,40] a client-centered interactional intervention in which the therapist adopts movements of every participant, creatively modifying them into related movement qualities with the group (“can we make this bigger, smaller, lighter, more direct, quicker, slower?” etc.) to provide a versatility and clarity in movement, inviting each participant to join in the movements offered, was conducted. Subsequently participants split into dyads and engaged in dyadic mirroring (about 15 to 20 min), each leading, following and then moving together freely but in constant contact with the same partner. Partners could be switched or remain the same over the sessions, this was left up to participants’ choice and coincidence. After that, the group came together again for the Baum-circle (about 20 min) [41] and mirrored movements of a volunteer participant to his/her own music while all are standing in a circle, with participants taking turns each to one entire self-selected song. Aims of the single movement interventions are compiled in Appendix B. Lastly, in the verbal processing part (about 10–15 min), experienced feelings related to self and others and evaluations and wishes were discussed under the lead of the therapist. The resulting verbal data was not collected and analyzed, since most verbal answers were stereotypic and flat, a typical phenomenon in ASD, whereas movement responses in the nonverbal parts of the sessions were rich, met the resources of the participants, and improved over the sessions (with video materials still under inspection/analysis). The procedure is manualized [35], however, necessary modifications due to disturbances or participants’ conditions can still be applied by the therapists during the sessions. Altogether six groups were conducted by two female trained dance movement therapist in three settings (one conducting four intervention groups in one setting and one conducting two intervention groups, one in each of two settings). Each group was accompanied by a student of DMT at SRH University Heidelberg as co-therapist and/or a psychology student from the University of Heidelberg, not previously known to the participants (10 female and two male students altogether). 

### 2.4. Clinical Assessment

Demographic data was assessed as part of a battery of questionnaires prior to and after the intervention period. Data on the primary outcome measure of the sub-study at hand (i.e., the level of negative symptoms) was acquired using the Scale for the Assessment of Negative Symptoms (SANS) [42]. Evaluation was carried out by clinical raters trained in the application of the scale. It consists of 24 items divided into five subsets constituting the scales (1) blunted affect (SANS-BA); (2) alogia (SANS-Al); (3) abulia/avolition (SANS-Ab); (4) anhedonia (SANS-Anh); and (5) diminished attention (SANS-Att). The global level of NS is assessed by a total score taking into account all items. Each item describes a symptom that is rated from absent (a score of 0) to severe (a score of 5). The SANS displays a satisfactory internal consistency (Cronbach’s α = 0.88) and external validity [43].

### 2.5. Sample

Power calculations revealed that 90 participants were required in order to detect a medium to large effect of 0.3–0.5 with a power of 0.8 and a two-tailed α of 0.5. Considerable drop-out was anticipated due to the relatively long time period of the study and the extensive assessment accompanied by motivational constraints. Taking this into account, and due to institutional conditions limiting the potential maximum of participants, a power of 0.8 was considered sufficient and recruitment aimed to comply with the required sample size as good as possible. 

Data on the SANS were collected from a total of *N* = 78 participants diagnosed with ASD according to the International Classification of Disorders—10th revision (ICD-10). The sample consisted of 12 women and 63 men (three did not provide data on gender) with a mean age of 22.5 years (standard deviation (SD) = 7.75, range 14–53). Most participants were German, lived without a partner, and had no children. Participants had no previous experience with DMT or similar therapeutic approaches. They were not familiar with either the therapist or the co-therapist/psychology student before the start of the sessions. See Table 1 for detailed information on the demographic data of treatment and control group respectively. The groups did not differ significantly in any of the demographic variables except for nationality (*χ*^2^(3) = 11.741; *p* < 0.01), which was only due to three non-German participants in the control group, and therefore does not impose any conceptual difficulties.

### 2.6. Statistical Analysis

#### 2.6.1. Missing Data

As expected, there was a large proportion of missing data regarding the SANS (22.4% in total), due to substantial drop-out. Only 43 participants provided data on the SANS for both pre- (T1) and post-test (T2). Out of the 35 participants with missing data, 18 did not provide data for the pre-test and 17 did not provide data for the post-test. If data was available for a measurement time, it was always complete for all subtests and thus also for the SANS total score. Because it is sufficient to assess demographics at one measurement point, missing data on the demographic data relevant for the analysis was negligible. Methods considered to successfully deal with the missing data were listwise deletion (LD) and multiple imputation (MI). In order to apply LD without obtaining biased results, data is required to be missing completely at random (MCAR); that is, missing values should not systematically differ from observed values. In order to apply MI, data is required to be at least missing at random (MAR), that is, any systematic difference between observed and missing data should be explained by one or more of the assessed variables, or MCAR [44]. In order to check the MCAR assumption, MCAR was tested by conducting Little’s Test which yielded non-significant results (*χ*^2^(8) = 8.07, *p* = 0.426). MAR was tested by conducting *t*-tests and *χ*^2^-tests on a variable that coded for missing values on the SANS and (1) group; (2) sex; (3) age; (4) SANS total score at T1 and (5) SANS total score at T2. Since none of the tests turned out to be significant, assumptions were considered to be met for both methods. In LD all participants with missing values at either T1 or T2 were excluded from the analysis, resulting in a smaller sample and thereby lower statistical power. In MI, data on missing values are imputed *m* times to create *m* complete data sets. After that, analyses are performed on each data set and the results are pooled. With this method, the obtained parameter estimates are less biased than by applying mean imputation [44]. Still, MI is not recommended for data sets with more than 20% of missing data (by convention) and, as LD does not produce biased results when the MCAR assumption is met, we chose to apply LD as the more conservative method to prevent artificially elevated power. 

#### 2.6.2. Main Analysis

Repeated measures ANOVA was applied to test for differences in the change of SANS-scores with *Group* (treatment versus control) as between-subject factor and *Time* (T1 versus T2) as within-subject factor. RM-ANOVAs were conducted for the SANS total score (SANS-TS) and for each subscore as follows: (1) SANS-BA; (2) SANS-Al; (3) SANS-Ab; (4) SANS-Anh; and (5) SANS-Att. To correct for alpha error-accumulation within the subscores, the Bonferroni-method (α = 0.05/*N*) was applied and the threshold was set to 0.01. 

## 3. Results

Regarding SANS, so far no meaningful cut-off values have been defined. Within the entire sample, SANS total scores ranged from 2 to 83 on a scale from 0 to 120 indicating “normal” to “severely ill” [45] with the sample mean (*M* = 32.97) slightly lower than “mildly ill”, named “borderline” to non-normal value. Psychometric properties of all outcome variables are displayed in Table 2 for the treatment and control group as well as for the entire sample and the complete cases subset respectively (including only those participants who provided data on both measurement times). There were no significant baseline differences between the groups in any of the outcome variables, within the entire sample, nor within the complete cases. Furthermore, means and standard deviations did not apparently differ between the entire sample and the complete cases subset. Therefore, and regarding the assumed MCAR structure (see Missing Data section above), results and conclusions drawn from the complete cases subset can be regarded as attaining to validity criteria. 

### 3.1. Changes in Severity of Overall Negative Symptoms

**Figure 1:**
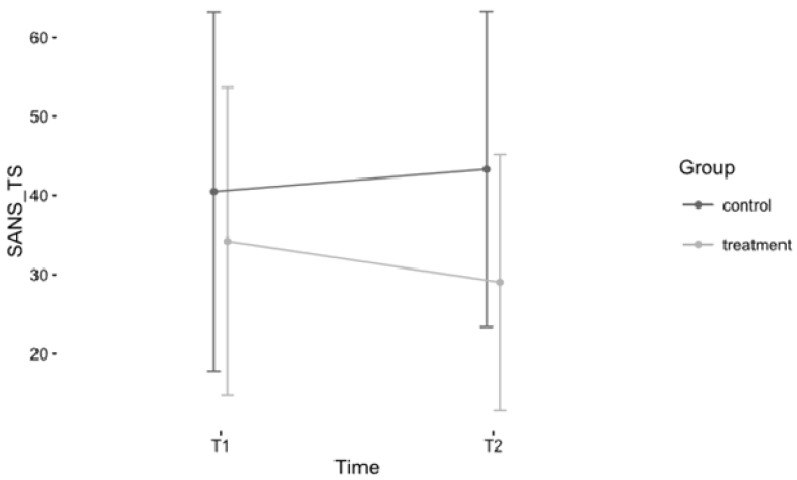
Development of SANS_TS scores for treatment and control group over time. Note: Error bars represent standard deviations; T1/T2 = measurement time one/measurement time two; SANS_TS, Scale for the Assessment of Negative Symptoms Total Score.

The SANS total score was not significantly related to any of the demographic variables we considered as potential confounds (age, gender and education), thus they were not included in the analysis. The repeated measures ANOVA of the SANS total score (SANS_TS), comprising the factors *Time* (T1, T2) and *Group* (treatment, control) revealed a marginally significant interaction effect on the significance level of 0.1 in the hypothesized direction indicating stronger symptom reduction in the treatment group (*F*(1, 41) = 2.99, *p* = 0.09). Furthermore, the main effect for *Group* was marginally significant (*F*(1, 41) = 2.95, *p* = 0.09), indicating higher values in the control group, while the main effect for *Time* was not significant. Effect sizes indicate a moderate effect for *Group* (probably due to baseline differences) and a small effect for the interaction term [46] (small effect: 0.01, thus at the borderline to very small). The mean symptom reduction in the treatment group was 15.27%, while symptom severity worsened by 6.99% in the control group. See Table 3 for detailed information on the results and Figure 1 for a graphic representation of the interaction of the factors *Time* and *Group* for the SANS total score.

### 3.2. Changes in Severity of Subdomains

Within the repeated measures ANOVAs on all subscores respectively, no interaction was significant at the 0.01 significance level (to which the threshold was set due to Bonferroni correction), nor at the 0.05 significance level. The significant main effects (on the 0.05 level) for *Group* and *Time* found in some subscales (see Table 4) were probably due to baseline differences and spontaneous overall symptom reduction and not relevant for our hypotheses. Although the interaction effects were not significant, information about the pattern of symptom development regarding the subscales can be drawn from the observed effect sizes. The strongest trends towards a higher symptom reduction in the treatment versus the control group were evident in the subscales “blunted affect” (SANS_BA) and “anhedonia” (SANS_Anh). All other subscales besides “alogia” (SANS_Al), in which symptom scores worsened in both conditions, followed this trend. See Table 4 for detailed information on the results of the repeated measures ANOVAs on all SANS subscales.

## 4. Discussion

### 4.1. Changes in Severity of Negative Symptoms

In the randomized controlled trial at hand we aimed to examine the effects of a manualized movement therapy intervention (based on DMT; [35]) on negative symptoms in participants with autism spectrum disorder. Relying on the embodiment approach and related research we hypothesized that participants receiving the DMT intervention should display a stronger decrease in negative symptoms over the period of the intervention compared to participants receiving no treatment. 

As expected, symptom reduction on the overall negative symptoms was greater in the treatment group, yet this effect was only significant at the 0.10 level and can thus be regarded as marginally significant (by convention). The observed symptom reduction of 15.27% is classified as a small effect and corresponds with a CGI-I (Clinical Global Impression-Improvement) score of “minimally improved”, which is regarded as clinically substantial [45]. In the light of the strong association of negative symptoms with poor quality of life and their extensive resistance to conventional treatment methods [5], a small improvement can be highly meaningful to the individual.

Participation in the treatment did not lead to a significantly different symptom reduction for any of the specific symptom subtypes. However, the symptom development pattern was consistent with the hypotheses for the NS subtypes anhedonia, blunted affect, abulia/avolition (ordered by decreasing effect size), and diminished attention (with a truly negligible effect size). Thus, symptom severity of these subtypes tended to decrease more in the treatment group and hence contributes to the effect observed for overall negative symptoms. Only with regard to alogia did symptom severity worsen in both conditions, however not significantly and with a truly negligible effect size (see Table 4). This pattern of response conforms to the predictions of embodiment theory, because the subtypes most affected by the intervention (anhedonia and blunted affect) are more closely related to diminished self-perception as the connection of bodily states to emotions, postulated to underlie the social deficits in autism spectrum disorder. 

As the intervention is mainly targeting the increase of empathy and perspective taking, and only to a minor degree of emotion expression, we would argue that the results are particularly encouraging. In future studies gearing the intervention more specifically to emotion expression, we would expect greater effects of DMT. DMT research at present is increasing its efforts to detect working mechanisms and active factors of specific interventions (e.g., [47,48]), which helps future studies in this research field.

The study at hand provides encouraging results and coincides with the benefits of dance movement therapy described in some case studies, for example, the increase of empathy and self-awareness in children with ASD [30]. Regarding quantitative approaches there are no studies with similar outcome variables, thus this study adds new insight to the limited quantitative evidence and research on DMT for young adults with ASD and demonstrates the far-ranging effects of DMT.

### 4.2. Limitations and Further Directions

Unfortunately, this study, as in many other studies working with longitudinal designs with clinical samples, there was a comparably high drop-out rate that led to two critical problems. First, a large amount of missing data needed to be accounted for. Although we carefully considered all possible methodic approaches and abided by statistical rules to avoid biased results, a great portion of valuable information was unavailable. This lead to the second problem: Due to the missing data, we could not comply with the required sample size of 90 participants to detect a medium to large effect with a power of 0.8 computed prior to the study. Taking the effect size found in this trial (generalized *η*^2^ = 0.009) as a basis, the required sample size to detect it would be even higher. 

Furthermore, several limitations regarding the treatment and the data acquisition are likely due to financial constraints. Firstly, and most importantly, the dyadic mirroring part of the intervention was predominantly performed in dyads of two participants rather than in dyads of a participant and a co-therapist. We learned from the comparison of the feasibility study and the main trial that, as the intervention aims to strengthen perception of others’ emotions (and thereby also perception of the self) by mirroring their movements, it seems to be crucial that the partner displays emotions in a non-autistic manner. If emotion expression is limited to the extent in which we find it in other participants with ASD, the effect seems to be strongly reduced. In the feasibility study for the present research project, dyadic mirroring was, barring rare exceptions, always performed with a co-therapist and treatment effects on all variables are stronger despite a smaller sample size [35]. 

Moreover, SANS ratings were conducted by trained student apprentices. In order to obtain reliable ratings, participation in more detailed workshops and a certain level of experience of the raters would be desirable. It can thus not be ruled out that raters in this study differed in their judgments, leading to unreliable ratings. Unfortunately, it was not possible to compute an inter-rater-reliability measure due to documentation. Hence, it was especially problematic that ratings on pre- and posttest of a participant were always conducted by different raters, while at the same time this was a good set-up for avoiding familiarity with tester as a confounding variable.

The use of a waiting control group is certainly a valid method to evaluate the effect of dance/movement therapy compared to the daily routine. Still, regarding the evaluation of embodiment approaches, it would be particularly interesting to examine whether advantages in symptom reduction are only due to general properties of physical and/or group activities or to specific properties postulated as effective by the embodiment notion. Future research should therefore focus on control interventions that differ from the movement therapy only in such aspects as for example, (1) the focus on mirroring; (2) the link of movement and self-perception; and (3) the link of movement and emotion. Another option would be to measure after each part of the intervention; e.g., after dyadic mirroring, after group mirroring with low exposure, and after group mirroring with high exposure; to be able to differentiate more effective from less effective interventions.

Lastly, the overall symptom severity in the autistic sample was merely at the threshold of non-normal values, which might be due to the fact that not all symptom subgroups of negative symptoms assessed with the SANS are typical for participants with ASD, and not all social symptoms relevant in ASD are measured by the SANS. This may have resulted in a floor effect on the SANS scores, which might partially explain why the observed effects were so small. Assessing the schizophrenia-typical symptom cluster of negative symptoms in autism was illustrative in order to compare effects of the intervention on participants with different disorders (see Appendix C). The comparison of the two populations showed that participants with ASD scored pronouncedly higher in negative affect values than participants from the schizophrenia spectrum (in a *Chi2*-test of the aggregated SANS Total Score Values over experimental group (EG) and control group (CG) for each diagnostic group the differences were significant at the *p* = 0.05-level; see Appendix C). Nonetheless, the use of ASD specific assessment instruments for the social symptom cluster would provide better and more comprehensive information about the relevant domains and thus be more serviceable in the search for truly effective treatment methods. 

## 5. Conclusions

The effect found in this study was significant at the 0.10 level, however, we observed an overall trend toward a stronger symptom reduction in almost all subtypes of NS and a small, yet clinically substantial effect size as judged by Levine and Leucht [45], equaling 15.27% of symptom reduction in overall NS. This is encouraging in the light of the low effectiveness of conventional treatment methods for ASD. Furthermore, the pattern of effect sizes of the symptom subtypes can be best explained by the embodiment approach as it matches the theoretic expectations on which subtypes should be most affected. The present study provides further evidence supporting the practice of body-oriented therapy forms with participants with ASD. The findings showed potentially influential factors on the decrease of negative affect, and thus the improvement of emotion expression, such as for example the importance of dyadic mirroring with a non-autistic partner, which future research can learn from and build upon.

## Figures and Tables

**Table 1:** Demographic data of the study participants.

Variable	Demographic Data	Treatment ^a^	Control ^b^
Gender	Female/Male/Missing **^c^**	9/44/2	3/19/1
Age	Means (years)/SD	23.07/8.54	21.27/5.32
Treatment Place	Bruchsal	8 (14.54%)	0
	Karlsruhe	14 (25.45%)	4 (17.39%)
	Ludwigshafen	31 (56.36%)	18 (78.26%)
	Missing	2 (3.64%)	1 (4.35%)
Nationality	German	41 (74.55%)	19 (82.61%)
	German + one additional	0	1 (4.35%)
	Other	0	2 (8.7%)
	Missing	14 (25.45%)	1 (4.35%)
First Language	German	39 (70.91%)	18 (78.26%)
	Other	2 (3.64%)	4 (17.39%)
	Missing	14 (25.45%)	1 (4.35%)
Education	No degree	3 (5.45%)	1 (4.35%)
	Hauptschule	9 (16.36%)	5 (21.74%)
	Realschule	7 (12.73%)	7 (30.43%)
	Abitur	6 (10.91%)	2 (8.7%)
	Completed formation	3 (5.45%)	2 (8.7%)
	Completed academic studies	2 (3.64%)	0
	Other	10 (18.18%)	4 (17.39%)
	Missing	15 (27.27%)	2 (8.7%)
Clinical Status	Day program	8 (14.5%)	4 (17.39%)
	Inpatient	1 (1.82%)	1 (4.35%)
	Outpatient	5 (9.09%)	1 (4.35%)
	Missing	41 (74.55%)	17 (72.91%)
Relationship Status	No partner	33 (60%)	15 (65.22%)
	Partner	3 (5.45%)	2 (8.7%)
	Missing	19 (34.55%)	6 (26.09%)
Martial Status	Single	34 (61.82%)	18 (78.26%)
	Wedded	1 (1.82%)	0
	Widowed	1 (1.82%)	0
	Divorced	2 (3.64%)	0
	Missing	17 (30.91%)	5 (21.74%)
Children	No children	34 (61.82%)	16 (69.57%)
	Children	2 (3.64%)	0
	Missing	19 (34.55%)	7 (30.43%)

Note: ^a^ *N* = 55; ^b^ *N* = 23; **^c^** SD= Standard Deviation.

**Table 2:** Psychometric properties of all outcome variables for the entire sample (**A**) and the complete cases (**B**).

(A) *N* = 78	Time of measurement	Treatment				Control			
(Maximal Value)		N	Means		SD^c^	N	Means		SD
SANS_TS	T1	45	33.51		19.27	15	37.93		20.89
−120	T2	41	28.37		15.63	20	37.45		18.94
SANS_BA	T1		13.6		7.47		12.93		9.15
−35	T2		9.78		6.36		12.65		9.15
SANS_Al	T1		3.87		3.72		5.27		4.7
−25	T2		3.63		3.52		5		4.41
SANS_Ab	T1		4.09		3.53		5.53		4.16
−20	T2		3.8		3.45		5.25		4.29
SANS_Anh	T1		8.67		5.14		9.13		5.6
−25	T2		7.66		4.41		9.8		3.94
SANS_Att	T1		3.73		2.96		5.07		3.26
−15	T2		3.49		2.98		4.75		2.79
**(B) *N* = 43**		**Treatment ^a^**				**Control ^b^**			
**(Maximal Value)**		**Means**		**SD**		**Means**		**SD**	
SANS_TS	T1	34.26		19.42		40.5		22.69	
−120	T2	29.03		16.2		43.33		19.92	
SANS_BA	T1	13.42		7.44		14.5		9.51	
−35	T2	10.03		6.83		14.67		9.16	
SANS_Al	T1	3.52		3.78		5.42		4.52	
−25	T2	3.48		3.59		5.75		4.97	
SANS_Ab	T1	4.53		3.96		6.17		4.2	
−20	T2	3.94		3.44		7.17		4.37	
SANS_Anh	T1	9.29		5.48		9.5		6.23	
−25	T2	8.13		4.49		10.75		4.11	
SANS_Att	T1	3.52		2.68		4.92		3.45	
−15	T2	3.1		2.77		5		2.66	

Note: SANS, Scale for the Assessment of Negative Symptoms; SANS_TS, SANS Total Score; SANS_BA, SANS subscale Blunted Affect; SANS_Al, SANS subscale Alogia; SANS_Ab, SANS subscale Abulia; SANS_Anh, SANS subscale Anhedonia; SANS_Att, SANS subscale diminished Attention; T1, measurement time one prior to the treatment period; T2, measurement time two after ten weeks of treatment or waiting; *n* equal in all variables. ^a^ *N* = 31, ^b^ *N* = 12, **^c^** SD = Standard Deviation.

**Table 3:** Results of repeated measures ANOVA for SANS total score.

Effect	*F*	*P*	*η*^2^
Group	2.95	0.093	0.058
Time	2.03	0.162	0.006
Group × Time	2.99	0.091	0.009

Note. *η*^2^ = generalized eta squared; degrees of freedom for effect = 1, degrees of freedom for residual = 41.

**Table 4:** Results of repeated measures ANOVAs for all SANS subscales.

Subscore	Effect	*F*	*P*	*η*^2^
(1) SANS_BA	Group	1.42	0.241	0.027
	Time	5.67	0.022 *	0.023 *
	Group × Time	2.51	**0.121**	**0.010**
(2) SANS_Al	Group	2.41	0.128	0.046
	Time	0.39	0.538	0.002
	Group × Time	0.00	0.993	0.000
(3) SANS_Ab	Group	04.5	0.04 *	0.077 *
	Time	0.06	0.809	0.000
	Group × Time	1.52	**0.224**	**0.008**
(4) SANS_Anh	Group	0.84	0.365	0.016
	Time	0.5	0.485	0.002
	Group × Time	2.44	**0.126**	**0.012**
(5) SANS_Att	Group	4.15	0.048 *	0.067 *
	Time	0.36	0.55	0.002
	Group × Time	0.24	0.629	0.002

Note: *η*^2^ = generalized eta squared; degrees of freedom for effect = 1, degrees of freedom for residual = 41. Threshold was set to 0.01 by Bonferroni method; effects significant at the 0.05 significance level are marked with an asterisk (*); effects with a relevant trend supporting the hypotheses are presented in bold.

## Appendix A

Poem of a female participant in the feasibility study for this clinical trial, reflecting her experience in dance/movement therapy with her friendly permission.

**We dance** Everything is dance, so one says. Even atoms swing and dance. Electrons circle around protons and neutrons. Everything swings, all is in harmony. Only this way the world is kept in an equilibrium It is an ancient law. And we also circle around each other in our dance. We dance, and the music animates us We dance, and the rhythms permeates us We dance, and find each other. We dance, and are joyful. We laugh and dance. We dance and are …free!

Note: A line of this poem was used in the title of the paper. The participant translated the German version of her poem into English herself. The poem was the result of a voluntary session assignment to the group of participants to reflect their experience in dance movement therapy (DMT) in another art medium, and bring it with them to the last session. The poem is a reprinted from the article “Fixing the mirrors” by Koch et al. [35]

## Appendix B

The general goal of the mirroring intervention for autism is the improvement of empathy, emotion expression and interpersonal interaction, particularly of natural/spontaneous nonverbal interaction skills. Moreover, this includes the improvement of nonverbal empathic skills, social competence, well-being, trust, body awareness, body image, and the distinction between self and other. The manualized DMT sessions consist of four parts, three predominantly nonverbal/movement parts and one predominantly verbal part. The intervention takes place in 7–10 weekly sessions of 60 min duration, led by a certified dance movement therapist and at least one co-therapist.

**Table B1:** Overview of the DMT Intervention “Therapeutic Mirroring for Autism” [35].

*Intro/Warm-up (approx. 10 min):* Unobtrusive mirroring in the circle. The *Chace-circle* is a low-threshold, high structure, client-centered warm-up technique that has proven beneficial for severely impacted client groups, with no contraindications reported [39]. It starts with free improvisation in movement to the music (“can everybody just take up the beat of the music and see how their body wants to move with it?”), where each person’s movement is taken on by the therapist and encouraged to be adopted and creatively explored by the group; participants are called upon with their name (“let’s all take the shoulders with it just like X”), movement is creatively explored, modifications are named (“can we enlarge quicken, slow down, make stronger the movement of X” etc.); the therapist also takes caution that everybody gets a full functional warm-up in the Chace-circle.	*Aim: “Setting the stage”* Initiation, reflection und variation in movement qualities and shapes; building *safety and trust* in space, therapist, and group as a pre-condition for authentic self-expression. The technique aims to *integrate* each participant, *make them feel seen* and feel part of the group, strengthens *group cohesion*, and fosters the *feeling of acceptance* and the development of the therapeutic relationship. The group is *coming together as a group*; it starts with the clients’ *nonverbal resources*, making his strength, autonomy, individuality and agency seen (empowerment; self-efficacy; invitation for emotional expression). Each person is seen and supported as s/he is (by naming them and reflecting their movement). *Relationship building; bodily grounding*; arriving at self and group; body awareness; *functional pleasure, extension of movement repertoire* and sensorimotor skills.
*Dyadic mirroring (approx. 15 min):* Two free choice partners are working together. To the first song, partner A leads in movement and partner B follows, to the second song B leads and A follows. “Follows” refers to mirroring of movement, reflecting rather the quality of movement (space, force, time) than its form/shape, to be with the partner respectfully and observantly (“mindfully”). In the third song “free improvisation” takes place, where both partners can engage in their own movements, but are asked to still relate to each other (“stay in contact”). On the basis of the free interaction part, observers can then rate/judge, whether the participant has a preference for concordant modal mirroring (oriented at the mirror axis; just as if standing in front of a mirror; e.g., A: left arm, B: right arm) or for concordant opposite mirroring (oriented at the own body axis; e.g., A: left arm, B: left arm); this allows the rater to assign them to a developmental stage of mirroring (corresponding to a mentalization stage) and to plan clinical interventions [23,24].	*Aim: Emotional empathy and interactional reciprocity through nonverbal dyadic sharing.* Via the joint practice of dyadic attunement (quality) and adjustment (shape) in (a) flexible roles/switches (leading and following) and (b) joint free improvisation (synchrony, differentiation, individuation, permeability (body as membrane to be employed in the service of our ability to resonate with each other), play, negotiating clash and repair, (c) empathic emotion is possible starting from movement synchrony; strengthening of sensorimotor skills, interactive, emotional and affective abilities, as well as general and interactional attention in the nonverbal dialogue. The nonverbal attuning/adjusting to the other; increase of permeability, flexibility and variability of expression and nonverbal reciprocity accompanied by anxiety reduction.
*Baum-circle (approx. 25 min):* This method starts in the big circle in the group setting [41]. One participant initiates authentic movements to an entire piece of music (“do your own thing to your music”; initially this is demonstrated exemplarily by the therapists/co-therapists). The music should be chosen and brought by the participant herself, election criterion should be personal (emotional) significance. The entire group follows the participant’s movements to this music in due consideration of the principles of attentive mirroring (“be with the person”). About four participants per session can lead one after the other.	*Aim: Emotional empathy and improved expression through nonverbal sharing in the group.* (a) *The initiating participant* strengthens his/her affective authentic expression to personal music; strengthens sensorimotor, attentional, emotional and communicative skills (including metaphor); improves communicability of internal arousal; (b) *The joining/following participants:* sense their bodily resonance with the music and the movement/expression of the initiator; by moving with the initiators, they start to note their feelings; kinesthetic empathy/sensorimotor attunement can lead to empathic emotion and cognition.
*Verbal processing (approx. 5–10 min *):* After the movement, the groups sits down and reflects the session (this can be aided by a work sheet). First, the initiating participant X reports on how s/he experienced moving to the music in the Baum-Circle (“was I able to express myself/what I wanted? What did it feel like that the others mirrored me?”), then the group members tell each other what it was like to move with X (“What did I feel? Was I able to relate?” etc.). Then, there is room to talk about the remaining parts of the session.	*Aim: Empathic emotion (and cognition/perspective taking) and improved expression/interaction through verbal sharing with the group.* Sharing of the subjective experience with focus on emotions in verbal feedback (emotional and cognitive empathy), empathic reactions and regulation between participants (emotional, cognitive, sensorimotor and social abilities); demarcation capability; perception of self and others; perception of the group and a safe space; reflection and feedback.

Note: * The verbal processing phase is usually longer, if working with other patient populations. In autism, the majority of participants is impaired by alexithymia and is thus limited in the ability to express sensations or emotions in words.

## Appendix C

**Table C1:** Comparison of psychometric properties of participants with autism spectrum disorders (ASD) of our study, and schizophrenic participants in the study of Martin et al., 2016 [36], both part of the TESIS project.

Outcome/Time	Treatment *N_S_* (T1 = 43; T2 = 30) *N_A_* (T1 = 45; T2 = 41)				Control *N_S_* (T1 = 21; T2 = 19) *N_A_* (T1 = 15; T2 = 20)			
	*M_S_*	*SD_S_*	*M_A_*	*SD_A_*	*M_S_*	*SD_S_*	*M_A_*	*SD_A_*
SANS-TS								
T1	28.72	*16.06*	33.51	*19.27*	17.48	*12.92*	37.93	*20.89*
T2	22.37	*12.11*	28.37	*15.63*	25.16	*14.43*	37.45	*18.94*
(1) SANS-BA								
T1	9.44	*6.14*	13.60	*7.47*	5.43	*7.26*	12.93	*9.15*
T2	7.19	*5.27*	9.78	*6.36*	8.26	*5.14*	12.65	*9.15*
(2) SANS-Al								
T1	3.00	*3.29*	3.87	*3.72*	1.43	*1.86*	5.27	*4.70*
T2	2.25	*2.63*	3.63	*3.52*	2.21	*3.24*	5.00	*4.41*
(3) SANS-Ab								
T1	5.05	*3.50*	4.09	*3.53*	4.43	*3.17*	5.53	*4.16*
T2	3.84	*2.41*	3.80	*3.45*	5.16	*4.32*	5.25	*4.29*
(4) SANS-Anh								
T1	6.88	*5.31*	8.67	*5.14*	3.81	*3.23*	9.13	*5.60*
T2	5.88	*3.82*	7.66	*4.41*	5.84	*4.54*	9.80	*3.94*
(5) SANS-Att								
T1	4.35	*2.84*	3.73	*2.96*	2.38	*2.13*	5.07	*3.26*
T2	2.63	*2.43*	3.49	*2.98*	3.68	*3.30*	4.75	*2.79*

Note: M_s_ = means for schizophrenic patients; SD_s_ = Standard Deviation for schizophrenic patients; M_a_ = means for participants with autism; SD_a_ = Standard deviation for participants with autism); N = number of participants; SANS, Scale for the Assessment of Negative Symptoms; SANS_TS, SANS Total Score; SANS_BA, SANS subscale Blunted Affect; SANS_Al, SANS subscale Alogia; SANS_Ab, SANS subscale Abulia; SANS_Anh, SANS subscale Anhedonia; SANS_Att, SANS subscale diminished Attention; T1 = measurement time one prior to the treatment period; T2 = measurement time two after 10 weeks of treatment or waiting; n of entire sample (not only complete cases); in a *Chi2*-test of the aggregated SANS Total Score values over experimental group (EG) and control group (CG) for each diagnostic group, the differences were significant with *p* < 0.05, with participants with ASD scoring higher on negative affect values.
